# Supplementary material for: Effect of disorder on transport properties in a tight-binding model for lead halide perovskites
Source: Sci Rep. 2017 Aug 21;7:8902. doi: 10.1038/s41598-017-09442-4 (PMC5566430; doi:10.1038/s41598-017-09442-4)
Supplement: Supplementary file 1 — Supplementary Information for: Effect of disorder on transport properties in a tight-binding model for lead halide perovskites [file 41598_2017_9442_MOESM1_ESM.pdf]

Supplementary Information for: Effect of disorder on transport properties in a tight-binding model for lead halide perovskites

S. Ashhab,[1] O. Voznyy,[2] S. Hoogland,[2] E. H. Sargent,[2] and M. E. Madjet[1]

[1]*Qatar Environment and Energy Research Institute (QEERI), Hamad Bin Khalifa University (HBKU), Qatar Foundation, Doha, Qatar*

[2]*Department of Electrical and Computer Engineering, University of Toronto, Toronto, Ontario M5S 3G4, Canada*

(Dated: June 20, 2017)

Here we present several additional results about the lead-halide perovskites obtained from the tight-binding model. Some of these results are closely related to our investigation of localization effects in disordered materials, while others are more general results pertaining to the tight-binding model of the lead-halide perovskites.

Rashba splitting

As mentioned in the main text, the band structure in Fig. 2 does not exhibit a Rashba splitting at the CBM and VBM because the Hamiltonian that are using in this work does not break inversion symmetry. We now show that if this symmetry is broken, a Rashba splitting is obtained. We modify the system parameters such that they are all kept unchanged except for the hopping strength between the Pb p orbitals and the I p orbitals when the I atom is below the Pb atom in the z direction, a change that makes the system not mirror symmetric about the xy plane anymore and hence breaks inversion symmetry in the z direction. The hopping between aligned p orbitals (i.e. σ configuration) is modified to 0.9 of the original value, and the hopping between parallel p orbitals (i.e. π configuration) is modified to 1.5 of the original value. We needed to make two parameter modifications in order to be able to independently adjust the Rashba splitting in the CB and VB: the former modification splits the bands at the VBM and CBM by equal amounts, while the latter modification contributes to the splitting at the VBM and causes a much smaller change at the CBM. The energy level spectrum around the R point, where the VBM and CBM are located, is plotted in Fig. 1.

Energy levels and IPR as functions of level index

In Fig. 2(a) we plot the energy levels of a $8 \times 8 \times 8$ supercell as a function of level index, in the absence of disorder and taking the quasimomentum to be zero. In other words, the energy levels obtained from diagonalizing the zero-quasimomentum, periodic-boundary-condition Hamiltonian are arranged in increasing order and plotted. Taking a large supercell and considering only zero quasi-momentum is in certain ways equivalent to taking a single unit cell but considering a large number of uniformly distributed quasi-momentum values. Indeed one can see that the DOS in Fig. 2(c) in the main text reflects the energy level

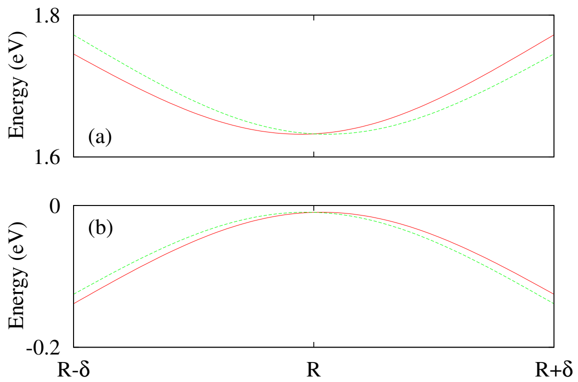

FIG. 1: Band structure of the tight-binding model with SOC and inversion symmetry breaking. We show only the energy levels at the CBM (a) and VBM (b), which are located at the R point. The range of the x axis is given by the parameter $\delta = (0.05, 0, 0)$. The single lines of the VBM and CBM in Fig. 2 of the main text are now split into two lines each.

distribution in Fig. 2(a).

We now include disorder in the on-site energies. In Fig. 2 we plot the energy levels as functions of level index for three different values of the disorder strength $\Delta\epsilon$. In Fig. 3 we plot the energy difference between the two states at the CBM (n=13,313) and VBM (n=13,312). For small values of $\Delta\epsilon$, up to around 0.2 eV, the on-site energy disorder has little effect on the energy levels. As $\Delta\epsilon$ is increased further and reaches the range 0.5-1 eV, the energy levels are modified drastically by the disorder and the energy gap disappears. If we increase $\Delta\epsilon$ further to 5 eV or higher, the energy levels form a distribution whose statistical properties reflect those of the Gaussian on-site energy fluctuations, hence losing all features that are specific to the underlying perovskite lattice. Note that the shrinking and eventual disappearance of the gap in the energy levels does not mean that the material will absorb photons of smaller frequencies and eventually become a conductor, because when we reach the point where the energy levels form a continuum the corresponding wavefunctions become separated spatially and one would not observe direct transitions between them.

In Fig. 4 we plot the IPR as a function of level index for three values of $\Delta\epsilon$. For small values of $\Delta\epsilon$, all the energy eigenstates are generally delocalized, although there are large variations in their IPR values. As $\Delta\epsilon$ is increased, the IPR generally increases and the states become more localized. Interestingly, this is not true for all the states, and some

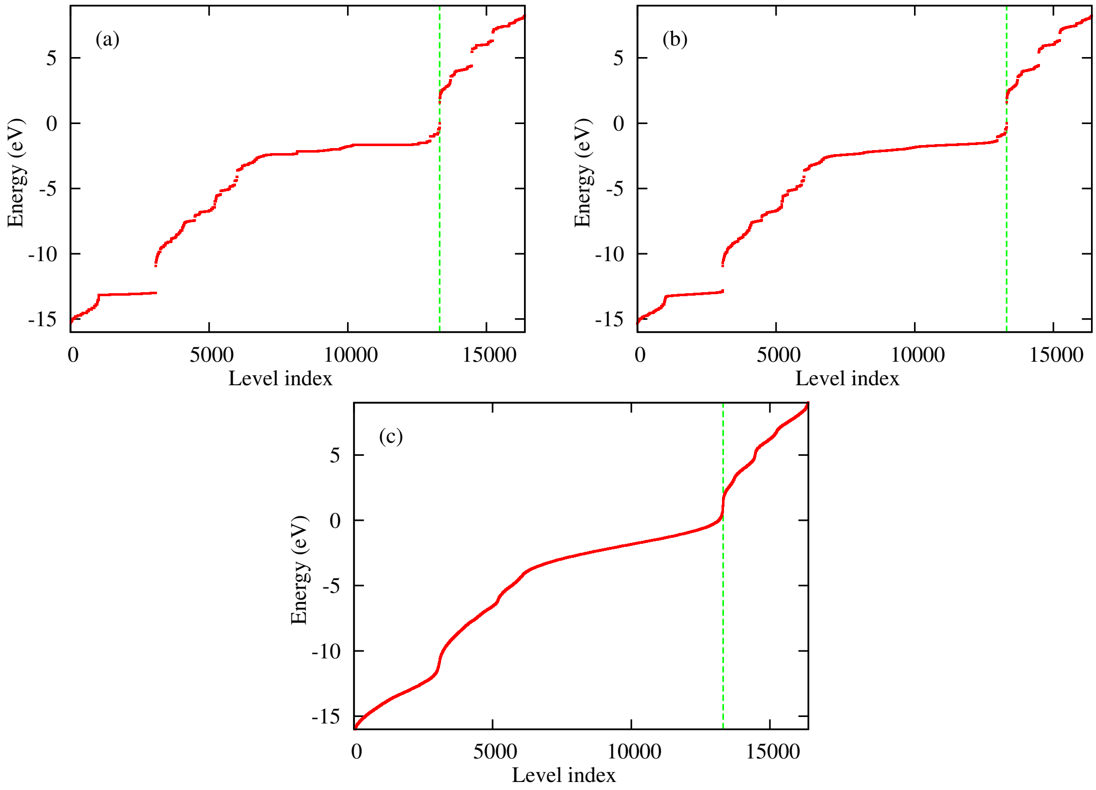

FIG. 2: Energy levels for an $8 \times 8 \times 8$ supercell at zero quasi-momentum as a function of energy level index when including disorder in the on-site energies. The disorder values used are $\Delta\epsilon = 0$ (a), 0.1 eV (b) and 1 eV (c). The energy level index ranges from 1 to 16,384, which is the number of electronic states in one supercell. The green vertical line marks the location of the VBM (n=13,312) and CBM (n=13,313). Panels (a) and (b) have another gap between the DVB (up to n=3,072) and the VB (starting from n=3,073). The figures plotted here are produced from single instances of the disordered potential, but we find that all the disorder instances that we have generated (i.e. five instances for each value of $\Delta\epsilon$) give results that look almost identical to those shown here.

states become more delocalized. This point is illustrated in Fig. 5(a), where we plot the ratio between the IPR at $\Delta\epsilon = 0.1$ eV and the IPR at $\Delta\epsilon = 0.01$ eV. The figure shows that the majority of the electronic states become more localized as $\Delta\epsilon$ is increased. However, some states, particularly near two energies inside the valence band, become less localized with increasing disorder. Another point to note in this figure is that the states around the VBM and CBM do not change drastically when the disorder is increased to 0.1 eV. Naturally, for very strong disorder all states become localized, as can be seen in Fig. 4(c).

We do not show the detailed plots of the IPR as a function of level index n, because it

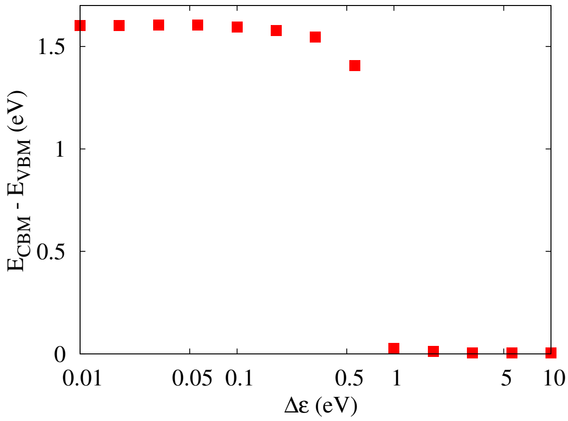

FIG. 3: The energy difference between level number 13,313 and level number 13,312 as a function of $\Delta\epsilon$. For small values of $\Delta\epsilon$, these two energy levels correspond to the CBM and VBM, respectively. The plotted quantity is therefore the band gap of the material. As can be seen in Fig. 2(c), for $\Delta\epsilon \gtrsim 1$ the band structure is lost. For such large values of $\Delta\epsilon$, the quantity plotted here loses its meaning as the band gap.

looks similar to that shown in Fig. 4(a). Similarly, the ratio between the IPR at $x = 0.5$ and the IPR at $x = 0$ as a function of energy level index is plotted in Fig. 5(b), and it exhibits similar features to those obtained in the case of on-site disorder.

Mixed-halide material band gap

In Fig. 6 we plot the band gap of the mixed-halide perovskite $MAPbI_{3(1-x)}Br_{3x}$ as a function of Br concentration x. The band gap increases almost linearly from 1.6 eV for $MAPbI_3$ to 2.3 eV for $MAPbBr_3$.

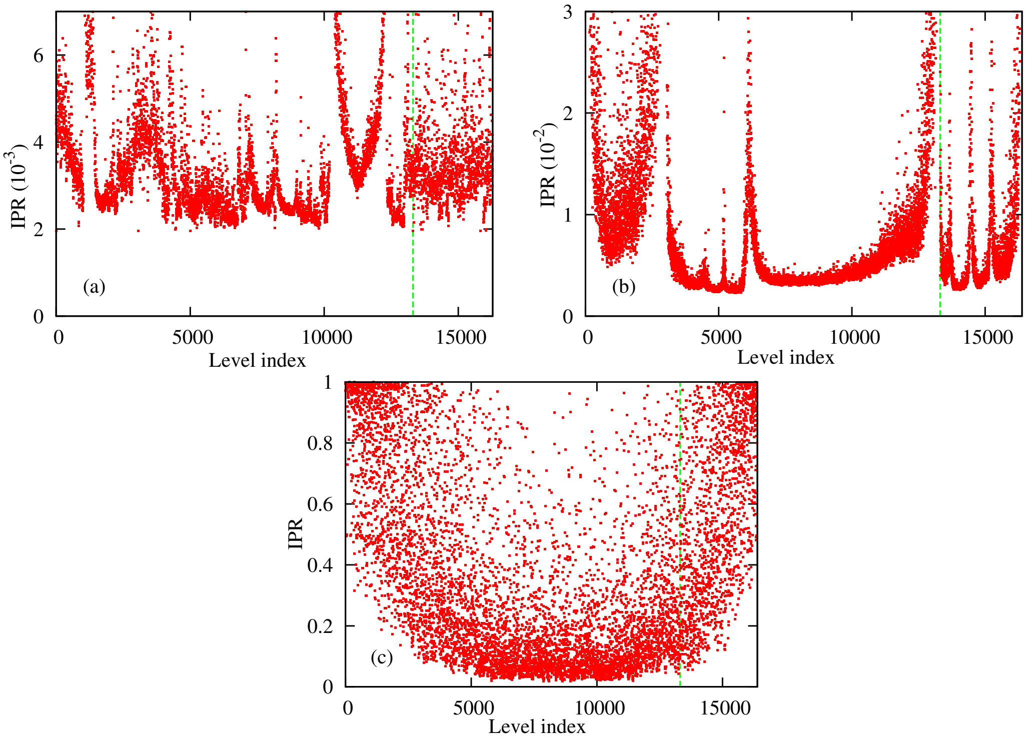

FIG. 4: Inverse participation ratio (IPR) for the energy eigenstates of an $8 \times 8 \times 8$ supercell as a function of energy level index in the presence of on-site energy fluctuations. The different panels correspond to different values of the disorder strength: $\Delta\epsilon = 0.01$ eV (a), 1 eV (b) and 10 eV (c). The green vertical line marks the location of the VBM and CBM. The figures are produced from single instances of the disordered potential, but we find that the overall features are the same for all five disorder instances that we have generated for each value of $\Delta\epsilon$.

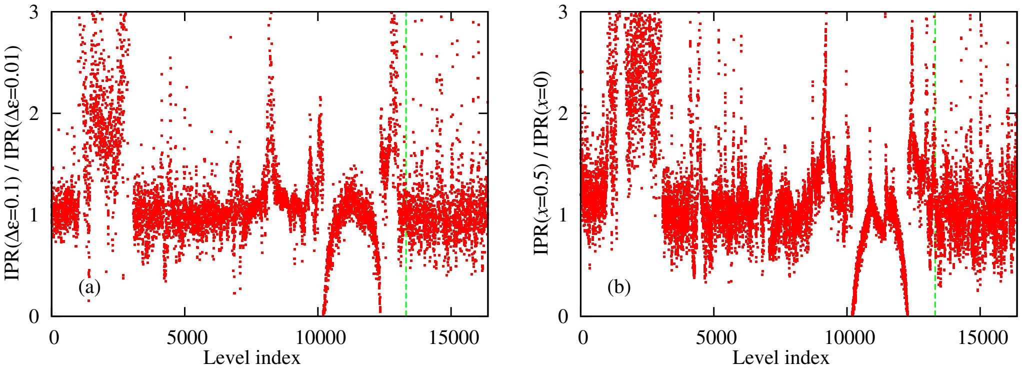

FIG. 5: The ratios IPR($\Delta\epsilon = 0.1$ eV)/IPR($\Delta\epsilon = 0.01$ eV) (Panel a) and IPR($x = 0.5$)/IPR($x = 0$) (Panel b). In (b) we use the model where Br orbital energies are different from those of I orbitals. The green vertical line marks the location of the VBM and CBM. This figures shows that some states become more localized while others become less localized when increasing the amount of disorder, keeping in mind that for very strong disorder all states become localized.

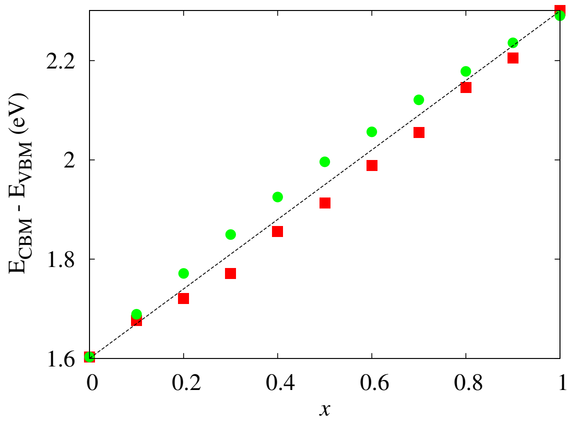

FIG. 6: Band gap as function of Br concentration x (where Br is treated as a dopant replacing I in $MAPbI_3$). The red squares and green circles correspond, respectively, to the models where the orbital energies and hopping strengths are modified when an I atom is replaced by a Br atom. The dashed line is a straight line the goes from 1.6 eV at $x = 0$ to 2.3 eV at $x = 1$. In both cases, the band gap increases almost linearly from 1.6 eV for $MAPbI_3$ to 2.3 eV for $MAPbBr_3$.
